# ICAM1 Is a Potential Cancer Stem Cell Marker of Esophageal Squamous Cell Carcinoma

**DOI:** 10.1371/journal.pone.0142834

**Published:** 2015-11-16

**Authors:** Sheng-Ta Tsai, Po-Jen Wang, Nia-Jhen Liou, Pei-Shan Lin, Chung-Hsuan Chen, Wei-Chao Chang

**Affiliations:** 1 Institute of Biochemistry & Molecular Biology, National Yang-Ming University, Taipei, Taiwan; 2 Graduate Institute of Cancer Biology, China Medical University, Taichung, Taiwan; 3 Center for Molecular Medicine, China Medical University Hospital, Taichung, Taiwan; 4 Genomics Research Center, Academia Sinica, Taipei, Taiwan; 5 Department of Chemistry, National Taiwan University, Taipei, Taiwan; 6 Institute of Atomic & Molecular Sciences, Academia Sinica, Taipei, Taiwan; China Medical University, TAIWAN

## Abstract

Esophageal squamous cell carcinoma (ESCC) accounts for about 90% of esophageal cancer diagnosed in Asian countries, with its incidence on the rise. Cancer stem cell (CSC; also known as tumor-initiating cells, TIC) is inherently resistant to cytotoxic chemotherapy and radiation and associates with poor prognosis and therapy failure. Targeting therapy against cancer stem cell has emerged as a potential therapeutic approach to develop effective regimens. However, the suitable CSC marker of ESCC for identification and targeting is still limited. In this study, we screened the novel CSC membrane protein markers using two distinct stemness characteristics of cancer cell lines by a comparative approach. After the validation of RT-PCR, qPCR and western blot analyses, intercellular adhesion molecule 1 (ICAM1) was identified as a potential CSC marker of ESCC. ICAM1 promotes cancer cell migration, invasion as well as increasing mesenchymal marker expression and attenuating epithelial marker expression. In addition, ICAM1 contributes to CSC properties, including sphere formation, drug resistance, and tumorigenesis in mouse xenotransplantation model. Based on the analysis of ICAM1-regulated proteins, we speculated that ICAM1 regulates CSC properties partly through an ICAM1-PTTG1IP-p53-DNMT1 pathway. Moreover, we observed that ICAM1 and CD44 could have a compensation effect on maintaining the stemness characteristics of ESCC, suggesting that the combination of multi-targeting therapies should be under serious consideration to acquire a more potent therapeutic effect on CSC of ESCC.

## Introduction

Esophageal cancer is the eighth leading cause of malignancies worldwide with its incidence on the rise [1]. It represents 1% of cancers diagnosed in the United States, with an estimated 17,500 new cases reported in 2012 [2]. Esophageal cancer is pathologically classified into two major subtypes, esophageal adenocarcinoma (EAC) and esophageal squamous cell carcinoma (ESCC). ESCC accounts for about 90% of esophageal cancer diagnosed in Asian countries. Since early detection strategies have not been well applied to clinical screen, these tumors are often diagnosed in advanced stages. Once metastasis occurs, cancer mortality significantly increases [3]. The overall 5-year survival rate after surgical resection is 70%~92% for patients without nodal involvement, but only 18%~47% for patients with lymph node metastasis [4]. The lack of fundamental knowledge regarding radiation and chemotherapy resistance in these tumor cells has been a major clinical barrier to acquire a better outcome. The limited understanding of the molecular biology of tumors has left us with empiricism in the clinic.

Cancer stem cell (CSC; also known as tumor-initiating cell, TIC) is defined as a subset of tumor cells with self-renew ability and involves in cancer initiation and progression. CSC is also highly resistant to radiation and chemotherapy and responsible for the cancer relapse after treatment [5]. Therefore, CSC has been viewed as an attractive target to destructively eliminate cancer cells. It is important to identify potential CSC markers that can be used to isolate CSCs and characterize their properties to be therapeutically targeted. Although CSC has been widely discovered in solid tumors, including breast, colon, glioma, prostate, liver, and melanoma [6–11], and multiple markers for their identification are available, the molecular marker for esophageal CSC is very limited.

Epithelial-to-mesenchymal transition (EMT) is an essential developmental process during mesoderm formation and neural tube formation, in which epithelial cells acquire a migratory mesenchymal phenotype [12]. The processes of tumor invasion and metastasis share many phenotypic similarities to EMT, including a loss of cell-cell adhesion and an increase in cell mobility. The link between CSC and EMT was first established in the transformed mammary epithelium [13], and the experimental results showed that TGFβ-induced EMT was associated with the acquisition of breast cancer cells with CD44^+^/CD24^-/low^ tumor-initiating phenotype, mesenchymal traits, and increased ability to form mammospheres. Recently acquired evidence indicated that CSC plays important roles in the metastasis of several types of carcinoma [14,15]. Therefore, increasing our general understanding of molecular biology of CSC will likely uncover the role of CSC in the metastasis of cancers.

Multiple integrated analyses, including genomics, epigenomics, transcriptomics, and proteomics, have been recruited to study the biology of CSC. Among them, proteomics holds a unique position in this area. For example, several major breakthroughs in CSC research were due to the identification of proteins using proteomic approach such as colony-stimulating factors [16] and cell-surface CD molecules [17]. Besides, proteomics is emerging as a powerful tool to identify the signaling complexes that control CSC differentiation and regulate CSC maintenance pathways [18]. A systematic proteomic approach to characterize CSC properties will shed new light on CSC biology and accelerate clinical applications in the prognosis, diagnosis, and therapy of cancer [19].

Membrane proteins, including enzymes, receptors, ion channels, and transporters, play many biological functions. Dysregulation of membrane proteins has been linked to a variety of human cancers [20]. Therefore, many membrane proteins have been characterized as markers for diagnosis and therapeutic targets, about 70% of existing pharmaceutical drug targets is membrane protein [21]. In this study, we aimed to identify potential surface markers of esophageal CSC using a proteomic approach. ESCC cell lines with various sphere formation ability were used for screening suitable surface markers. The CSC properties of potential marker were further characterized using colony formation assay, drug resistant assay, and tumorigenicity assay in immune deficient mice.

## Materials and Methods

### Cell lines and Cell culture

In this study, human esophageal cancer cell lines were purchased from the Bioresource Collection and Research Center of Food Industry Research and Development Institute (Hsinchu, Taiwan; http://www.bcrc.firdi.org.tw/). Cell lines CE81T/VGH (CE81T; BCRC 60166) and CE146T/VGH (CE146T; BCRC 60167) were derived from 57-, and 50-year-old Taiwanese males, respectively. Both cell lines were cultured in Dulbecco’s modified Eagle’s minimal essential medium (DMEM; Gibco BRL) supplemented with 10% (v/v) fetal bovine serum (FBS) (Gibco BRL), 100 U/ml penicillin, and 100 mg/ml streptomycin (Gibco BRL), and kept at 37°C in a 5% CO_2_/95% air atmosphere.

### Membrane protein extraction and in-gel digestion

The compartmental protein extraction kits CNM (BioChain Institute) was used to extract membrane proteins of cancer cells. The CNM kit includes two chemical extraction steps: (1) removal of cytoplasmic and nucleic proteins by reaction buffer containing HEPES, MgCl_2_, KCl, sucrose, glycerol, and sodium orthovanadate; (2) extraction membrane proteins by NP40 and sodium deoxycholate. Before mass spectrometer analysis, membrane proteins were separated by SDS-PAGE and divided into ten gel fractions, which were then cut into small gel pieces (<1mm^3^) and subjected to in-gel digestion, individually. The in-gel digestion procedure includes coomassie blue destaining, disulfide-bond reduction, acrylation with iodoacetamide, and trypsin digestion, followed our previously described method [22].

### Matrigel invasion and wound healing assay

*In vitro* invasion was analyzed using Matrigel-coated (1 mg/ml; BD Biosciences) PET membrane transwell insert (BD Biosciences) on a 24-well plate. Cancer cells (1.5×10^5^ cells in 200 μl) were suspended in DMEM medium and added to the upper half of the insert chamber. DMEM medium supplemented with 10% FBS was added as a chemoattractant to the lower half. After incubation at 37°C for 24 hr, cancer cells passed through the insert were fixed with 3.7% formalin (Sigma-Aldrich) and stained with 0.1% crystal violet (Sigma-Aldrich).

*In vitro* migration was analyzed using ibidi Culture-Insert (ibidi). Cancer cells (70μl; concentration: 7x10^5^ cells/ml) were added to Culture-Insert well and cultured for 24 hr. After removal of Culture-Insert, cancer cells were cultured for 16 hr. The migration distance of cancer cells was recorded and measured using software image J.

### Sphere formation assay

Sphere formation assay was performed in 6cm culture dish coated with 1% agarose. Cancer cells suspended in serum free medium were seeded at a density 5,000 cells/dish and incubated for 7 days. The numbers of primary sphere were counted manually at 7th day under microscope. For secondary sphere assay, spheres from primary culture were collected and incubated with trypsin at room temperature for 10 min. Trypsinized cells from spheres were passed through 26 G needle three times. Dissociated cells were individually plated at 5,000 cells/dish density in 6cm culture dish for 7 days. The numbers of secondary sphere were counted at 7th day.

### Tumorigenicity assay in immune deficient mouse model

The animal procedure (102-97-N) was approved by the Institutional Animal Care and Use Committee (IACUC) at China Medical University Hospital (Taichung, Taiwan). Various doses of CE146T cells (10^2^, 10^3^, and 10^4^) were injected subcutaneously into both flanks of 5-week-old male NOD-SCID mice to generate tumors. Left flank was injected with control group of cancer cells, and right flank was injected with experimental group. Tumor size was measured by caliper measurement weekly when tumors became visible, and the duration of observation was eight weeks. Tumor volume was calculated with the formula: (length x width^2^)/2.

### Mass spectrometer analysis

Mass spectrometer analysis was performed using the linear ion trap-Fourier transform ion cyclotron resonance mass spectrometer (LTQ-FTICR MS; Thermo Fisher) equipped with a nano-electrospray ion source (New Objective) and a nano-HPLC system. Nano-HPLC separation used a reverse nano-column (75 μm I.D. × 200 mm) packaged with Magic C18AQ resin (particle size 5 μm, pore size 200 A°; Michrom Bioresources) and an Agilent 1100 series binary HPLC pump (Agilent Technologies). The analytic program was set at a linear gradient from 10% to 50% ACN with a 60 min running cycle. The survey scan of MS analysis (*m/z* 320–2,000) was performed in LTQ-FTICR MS with a mass resolution of 100,000 at *m/z* 400. Top ten most abundant multiply charged ions were sequentially isolated for MS/MS by LTQ. The resulting data were applied to the MaxQuant software [23] for protein identification. Accurate label-free quantification was performed using the MaxLFQ program by normalization and maximal peptide ratio extraction methods [24]. The significance threshold for the identification was set to *P* < .01.

### Statistics

Data were expressed as means ± SD. The significance of difference was examined by Student’s *t*-test (two-tailed). *P* < 0.05 was considered to be significant.

## Results

### CE146T has more metastatic potential and stemness characteristics than CE81T

**Fig 1:**
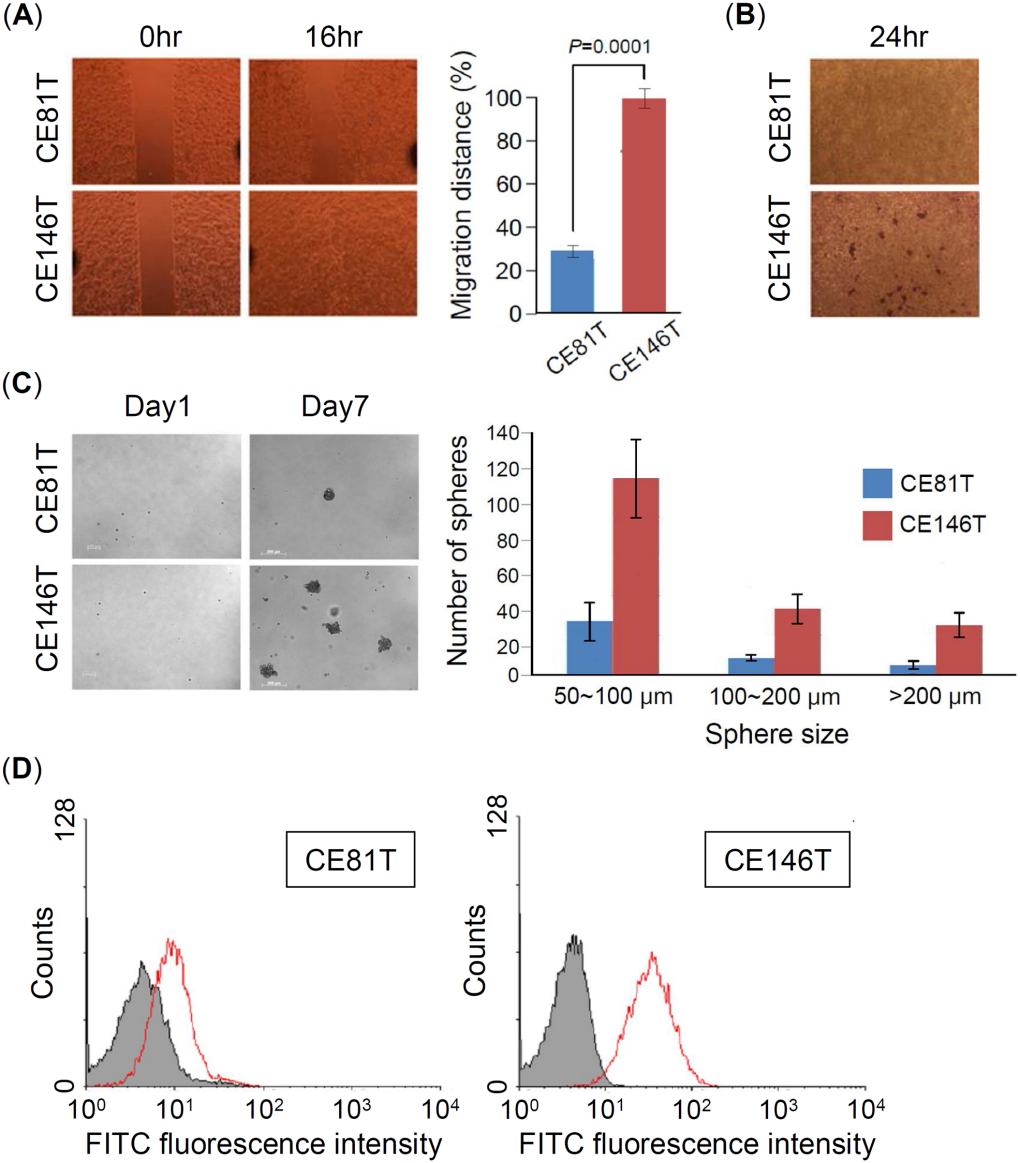
CE146T has more metastatic potential and stemness characteristics than CE81T. (A) Wound-healing assay. CE81T and CE146T were cultured in the ibidi Culture-Insert well, and the migration images of cancer cells were recorded at 0hr (removal of Culture-Insert) and 16hr. Migration distance was measured using software image J. (B) Matrigel invasion assay. Representative photographs contain invaded cancel cells that were stained with crystal violet. (C) Sphere formation assay. Various sizes of spheres in CE81T and CE146T were counted at day 7. Experiments were performed in triplicate (mean ± SD). (D) CD44 expression in CE81T and CE146T was measured using flow cytometry.

To choose suitable target cell for identification of novel CSC markers, we first characterized the metastatic properties such as migration and invasion and sphere formation ability of esophageal squamous cancer cell lines CE81T and CE146T. The results showed that CE146T has remarkably higher migration and invasion abilities than CE81T (Fig 1A and 1B), which was identified as a well differentiated cancer cell. In addition, CE146T also has higher sphere formation ability than CE81T (Fig 1C). Recent studies demonstrated that CSC of ESCC expresses high levels of CD44 [25,26]. Accordingly, we used flow cytometry to analyze the expression of CD44 in CE146T and CE81T. The result showed that CE146T has higher levels of CD44 than CE81T (Fig 1D). Taken together, these data indicated that CE146T has more CSC population and exhibits more CSC properties than CE81T, implying that we are able to identify CSC markers by comparing the proteomic changes of CE146T and CE81T.

### Identification of CSC markers using a comparative membrane proteomic approach

Membrane protein has the advantage in diagnostic and therapeutic application. Thus, we used a comparative membrane proteomic analysis to identify the novel CSC markers from CE146T and CE81T. Although CE146T and CE81T are not isogenic, CE146T may express higher levels of proteins associated with CSC properties than CE81T based on above observations. In MS analysis, two technical replicates were performed for each sample. Protein identification and label-free quantification of mass signals were analyzed using the MaxQuant software [23]. In this analysis, total 652 membrane proteins were identified, which included 266 plasma membrane proteins based on Gene Ontology definition. Among membrane proteins, total 13 proteins showed specific expression or more than a 50-fold increase of expression in CE146T compared with CE81T (Table 1).

**Table 1:** Up-expressed membrane proteins in CE146T compared with CE81T.

Uniprot	Protein Name	Gene Name	CE146T/CE81T	M.W. [kDa]	Unique #	PEP ^(a)^
P30481	HLA class I histocompatibility antigen, B-44 alpha chain	HLA-B	>694 ^(b)^	47.95	13	7.66E-196
P08195	4F2 cell-surface antigen heavy chain [CD98]	SLC3A2	>672	67.99	35	<1.0E-307 ^(c)^
Q8WU90	Zinc finger CCCH domain-containing protein 15	ZC3H15	>176	49.14	2	1.70E-05
Q15942	Zyxin	ZYX	>163	61.28	3	1.32E-06
Q8N4V1	Membrane magnesium transporter 1	MMGT1	>148	21.88	2	7.28E-15
Q6IAA8	Ragulator complex protein LAMTOR1	LAMTOR1	>57	17.75	2	1.40E-21
Q9BTU6	Phosphatidylinositol 4-kinase type 2-alpha	PI4K2A	>24	54.02	3	1.15E-08
P51151	Ras-related protein Rab-9A	RAB9A	>21	22.84	3	2.81E-19
Q9NUP9	Protein lin-7 homolog C	LIN7C	>21	21.83	2	2.18E-06
Q8N271	Prominin-2	PROM2	>16	91.88	2	5.36E-02
P05362	Intercellular adhesion molecule 1 [CD54]	ICAM1	105	57.83	11	2.77E-84
P18462	HLA class I histocompatibility antigen, A-25 alpha chain	HLA-A	77	41.62	15	2.37E-293
O95832	Claudin-1	CLDN1	61	22.74	2	2.46E-30

^(a)^ Posterior error probability (PEP) was obtained from statistical analysis of total peptide identification for a protein in one sample. The value essentially operates as a statistical value, and low PEP indicates high statistical significance.

^(b)^ The symbol > represents the mass intensity in CE81T is under detection limitation and its value is set as threshold count 10,000.

^(c)^ The symbol < represents the PEP value is below 1x10^-307^ that is the lowest limit of Excel table.

**Fig 2:**
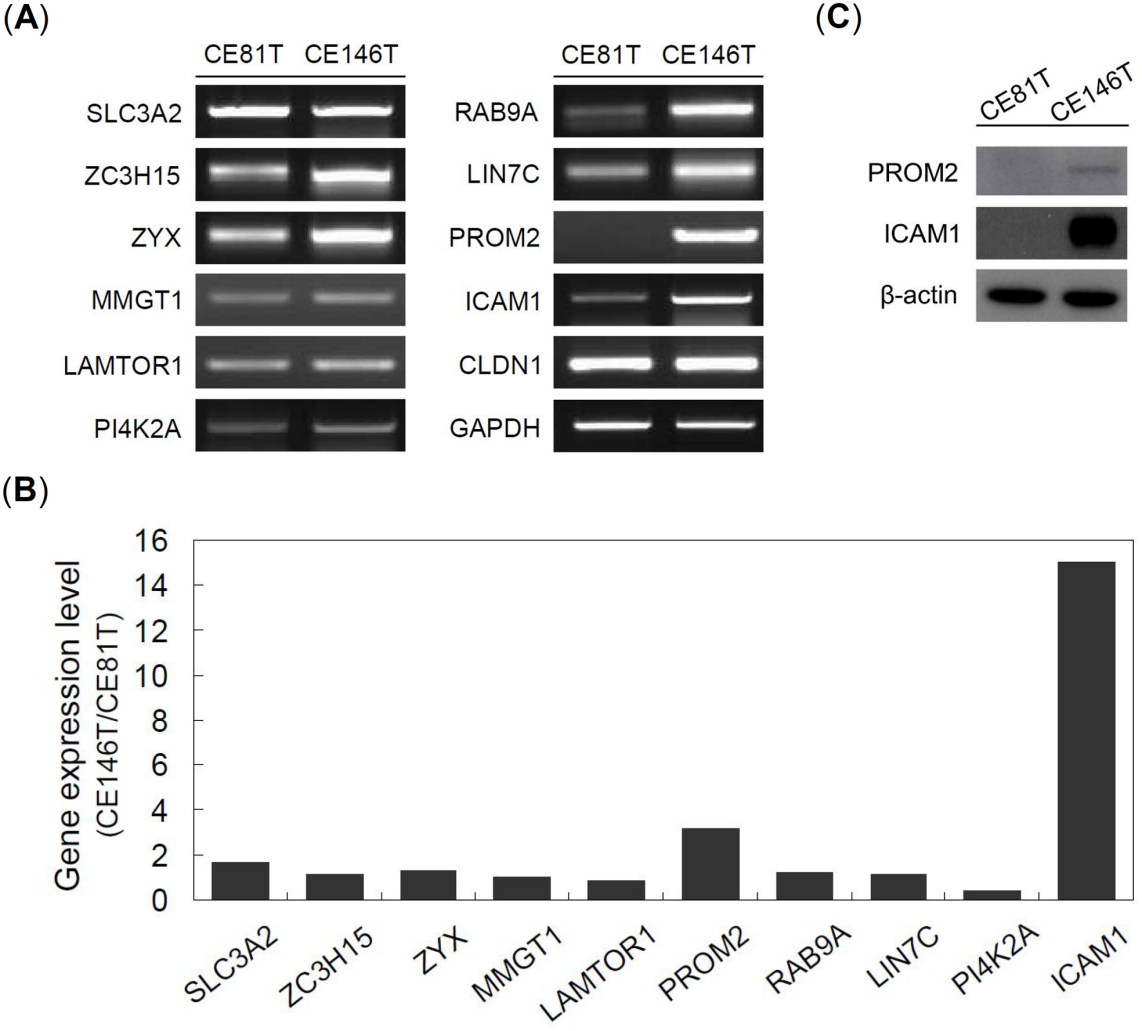
Validation of the proteomic data. (A) The mRNA levels of target proteins were analyzed using RT-PCR assay. GAPDH served as control. (B) The mRNA levels of target proteins were analyzed using qPCR assay. (C) The protein levels of both PROM2 and ICAM1 were analyzed using western blot assay. β-actin served as loading control.

To validate MS results, we used RT-PCR, quantitative PCR (qPCR), and western blot to examine the gene and protein expression levels of candidate proteins except two histocompatibility antigens. RT-PCR and qPCR results showed that intercellular adhesion molecule 1 (ICAM1) and prominin-2 (PROM2) have higher gene expression in CE146T than in CE81T, which is consistent with the proteomic finding (Fig 2A and 2B). Western blot showed that ICAM1 has a high expression in CE146T, but not PROM2 (Fig 2C). Accordingly, we further evaluated the character of ICAM1 on CSC properties of ESCC.

### ICAM1 increases metastatic potential of cancer cells

**Fig 3:**
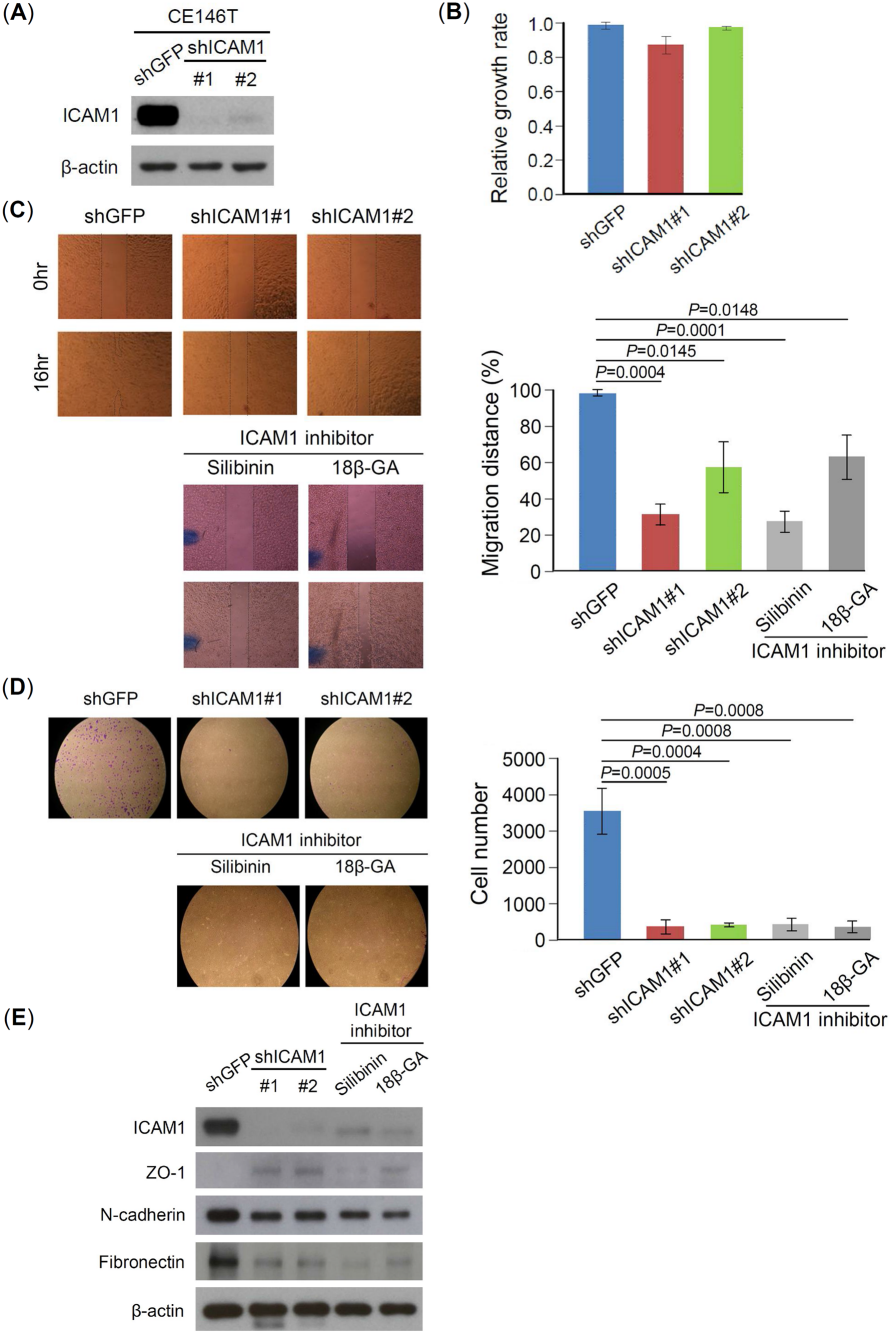
ICAM1 increases metastatic potential of cancer cells. (A) ICAM1 expression was knocked down using two kinds of shRNA, shRNA#1 target sequence is: GCCCGAGCTCAAGTGTCTAAA, and shRNA#2 target sequence is: CCTCAGCACGTACCTCTATAA. (B) Cell growth of CE146T was analyzed using MTT assay. ICAM1 knockdown did not significantly affect the growth of CE146T cells. CE146T-shGFP served as control. The effects of ICAM1 knockdown on cell migration and invasion abilities were analyzed using (C) wound-healing and (D) matrigel invasion assays, respectively. Both experiments were performed in triplicate (mean ± SD). (E) The expression of EMT markers was determined using western blot assay. CE146T was treated with ICAM1 inhibitor silibinin (2μM) or 18β-GA (10μM) for 24hr. The expressions of epithelial marker ZO-1 and mesenchymal markers N-cadherin and fibronectin were examined using specific antibodies. β-actin served as loading control.

To evaluate the functions of ICAM1 on CSC properties, we used the lentiviral vector expressing ICAM1-specific short hairpin RNA (shRNA) to knock down ICAM1 expression. Two kinds of ICAM1 shRNA were transfected into CE146T (labeled as shICAM1#1 and shICAM1#2), both transfected cells showed obvious knockdown of ICAM1 in western blot assay (Fig 3A). ICAM1 knockdown did not affect cell growth of CE146T (Fig 3B), but it significantly reduced cell migration and invasion abilities of CE146T in wound-healing and transwell assays, respectively (Fig 3C and 3D). In addition, two pharmacological ICAM1 inhibitors, silibinin [27] and 18 beta-glycyrrhetinic acid (18β-GA) [28], displayed similar effect on reducing the migration and invasion abilities of CE146T (Fig 3C and 3D). The results indicated that ICAM1 contributes to CE146T metastatic properties *in vitro*. Therefore, we further determined whether ICAM1 knockdown affects the expression levels of EMT markers. Western blot result showed that both shRNA and pharmacological inhibition could increase the levels of epithelial marker such as ZO-1 and attenuate the levels of mesenchymal markers such as N-cadherin and fibronectin (Fig 3E).

### ICAM1 increases sphere formation and drug resistance

**Fig 4:**
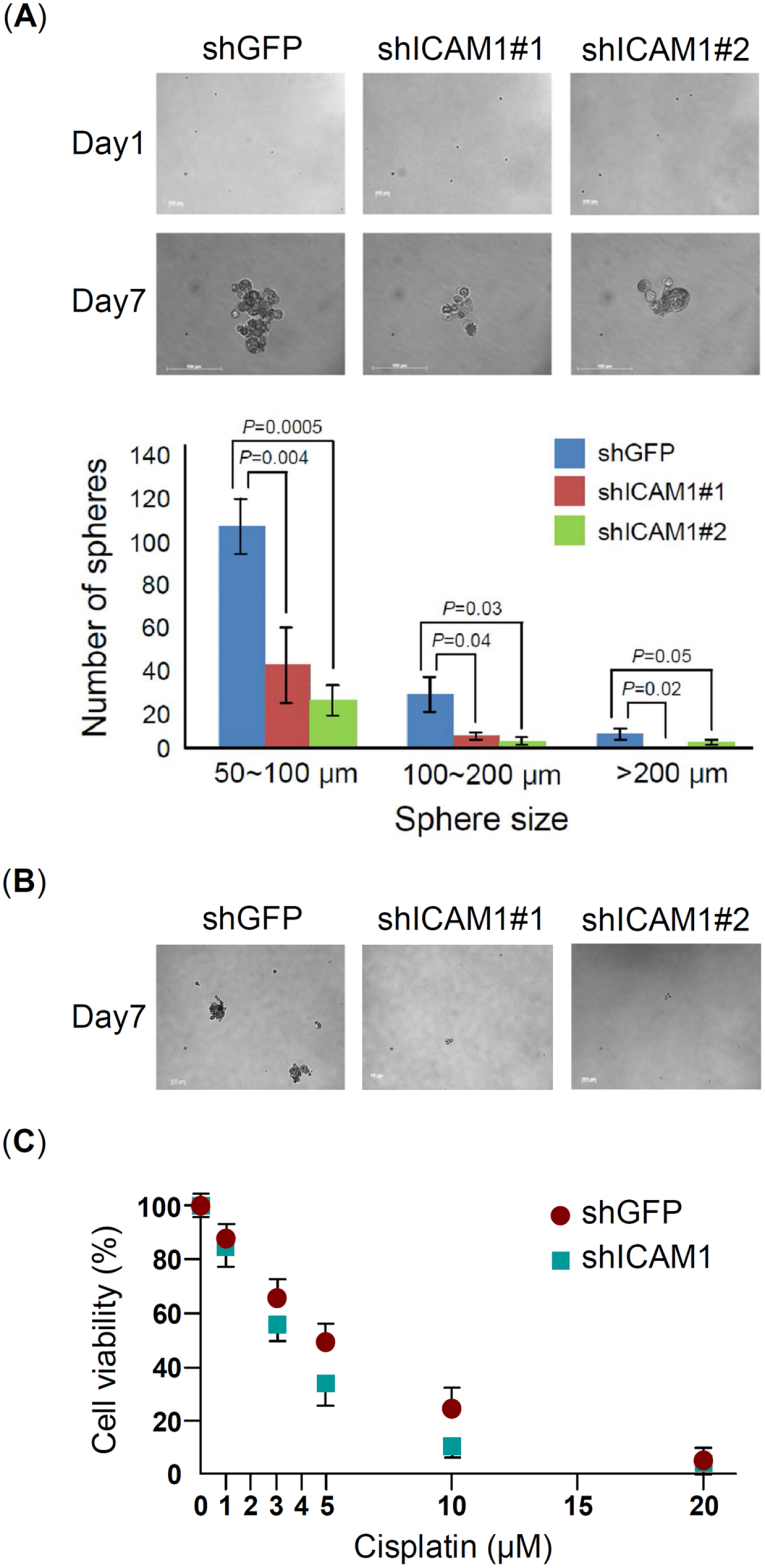
ICAM1 increases sphere formation and drug resistance of CE146T. The assays of (A) primary sphere formation and (B) secondary sphere formation were performed in CE146T with or without ICAM1 knockdown. Sphere number and size were observed and counted of primary at 7th day culture. (C) Drug resistance analysis. CE146T-shGFP and ICAM1-shICAM1#1 were treated with various doses of cisplatin for 24hr, and cell viability was measured using MTT assay. Each experiment was performed in triplicate (mean ± SD).

CSC has been known to increase the ability of sphere formation and express highly resistant to radiation and chemotherapy. To character the functions of ICAM1 on sphere formation, we compared the ability of sphere formation between shICAM1#1 and shICAM1#2 and their control group shGFP of CE146T. The result indicated that ICAM1 knockdown significantly reduces the sphere formation ability of CE146Y, no matter in sphere number or in sphere size (Fig 4A). In addition, it also significantly reduced the secondary sphere formation of CE146T (Fig 4B).

Cisplatin is a commonly used cytotoxic agent in chemotherapy of esophageal cancer, while cisplatin has also a clinically important radio-sensitizing effect that makes combination therapy practicable [29]. To evaluate the functions of ICAM1 on cisplatin resistance, we treated CE146T with various doses of cisplatin and determined cell viability using MTT assay. The result showed that ICAM1 knockdown reduces the ability of cisplatin resistance of CE146T, and the IC_50_ of CE146T in control group (shGFP) and ICAM1 knockdown group (shICA1#1) are 5 μM and 3.5 μM of cisplatin, respectively (Fig 4C).

### ICAM1 promotes tumorigenesis in immune deficient mice

**Fig 5:**
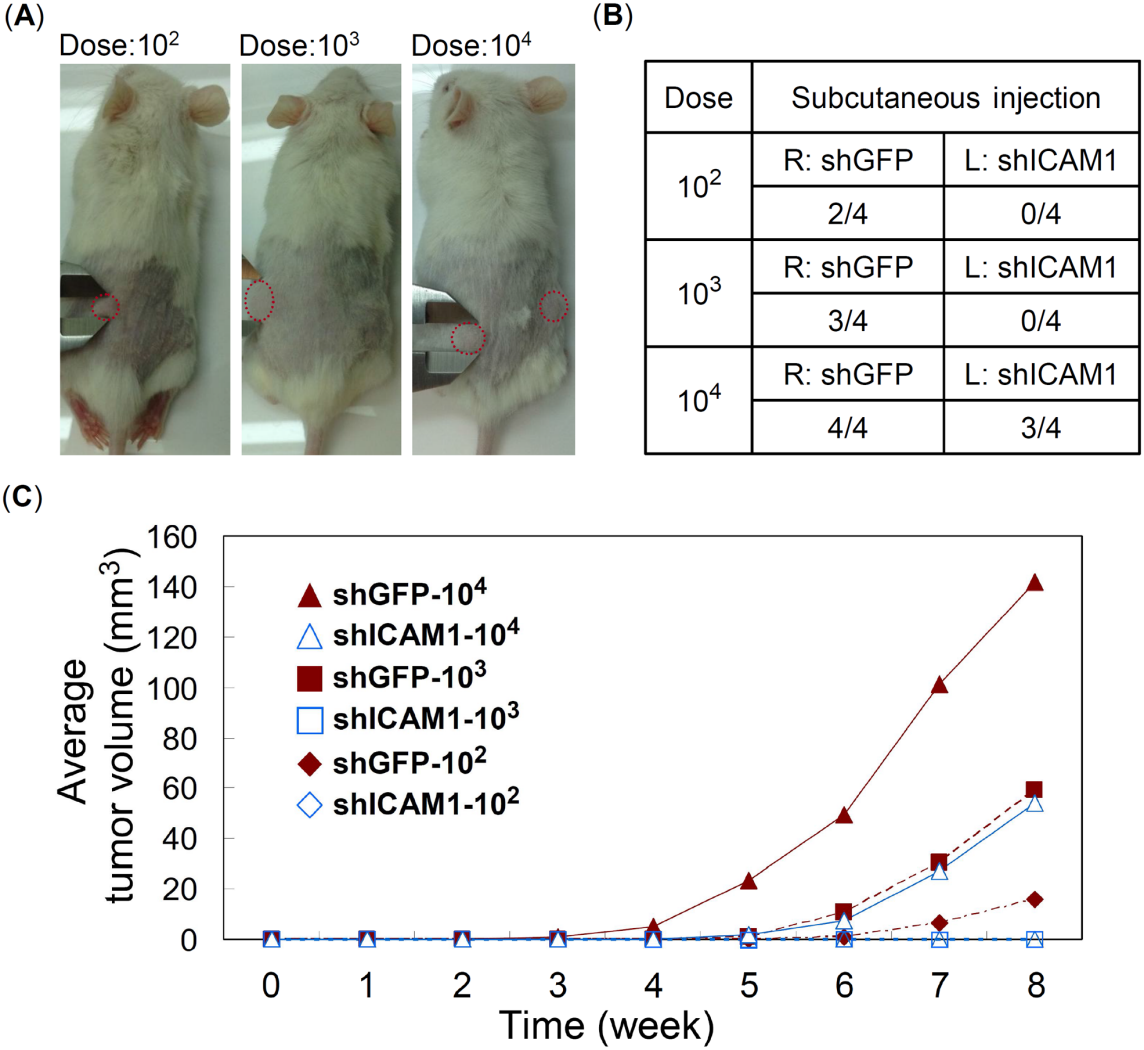
ICAM1 increases tumorigenesis of CE146T. (A) Various doses of CE146T cells, 1x10^2^, 1x10^3^, and 1x10^4^, were injected subcutaneously into both flanks of 5-week-old male NOD-SCID mice (*N* = 4). Representative subcutaneous tumors derived from CE146T-shGPF in left flank and CE146T-shICAM1#1 in right flank. (B) The number of tumor formation in each group was recorded and summarized. (C) Tumor size was recorded weekly and the average tumor volume of each group was plotted. The observation was continued for eight weeks after inoculation.

To study the functions of ICAM1 on tumorigenesis *in vivo*, we injected subcutaneously into both flanks of NOD-SCID mice with 10^2^, 10^3^, or 10^4^ of CE146T cells. Left flank was injected with control group of CE146T (shGFP), and right flank was injected with experimental group of CE146T (shICAM1#1) (Fig 5A). The results showed that even low dose of CE146T cells (10^2^) without subpopulation enrichment is able to induce tumorigenesis in NOD-SCID mice, suggesting that CE146T indeed has high population of CSC (Fig 5B and 5C). In addition, higher dose of ICAM1 knockdown CE146T cells (10^4^) are needed to induce tumor formation, revealing that ICAM1 knockdown reduces *in vivo* tumorigenic potential, one of the important properties of CSC (Fig 5B and 5C).

### ICAM1 regulates esophageal CSC properties partly through p53-dependent pathway

**Fig 6:**
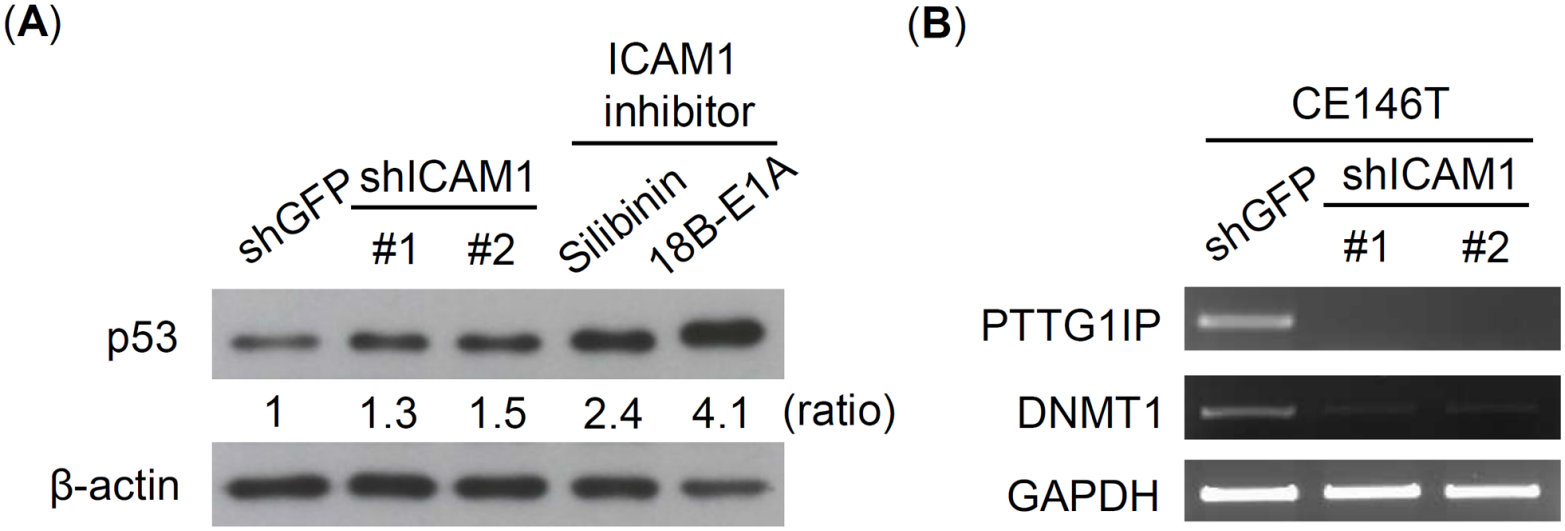
ICAM1 affects the expression levels of p53 and p53-related proteins. (A) The p53 levels of CE146T in shGFP control and under shRNA knockdown or ICAM1 inhibitor conditions were analyzed using western blot assay. The relative ratio of p53 levels was normalized with the β-actin levels. (B) The mRNA levels of p53-related proteins PTTG1IP and DNMT1 were analyzed using RT-PCR assay. GAPDH served as control.

To realize the potential mechanism of ICAM1 in regulating CSC properties, we performed a comparative proteomic analysis to investigate the protein change between shGFP group and shICAM1 group of CE146T. Among the identified list (S1 Table), we noticed a p53-related subgroup significantly down-expressed along with ICAM1 knockdown (Table 2). The p53 is a well studied tumor suppressor in cancer biology. Recent studies in the stem cell field have highlighted a profound role of p53 as the barrier to cancer stem cell formation [30,31]. Therefore, we examined p53 protein levels of CE146T in shGFP control and under the treatment of shRNA or pharmacological ICAM1 inhibitor condition. Western blot revealed that ICAM1 knockdown and pharmacological inhibitors are able to increase p53 levels of CE146T (Fig 6A), implying that ICAM1 may regulate esophageal CSC characters, at least partly, by reducing p53 levels and its related signaling pathways. We further validated the p53-related subgroup of proteomic finding using RT-PCR assay. The result showed that mRNA levels of pituitary tumor-transforming gene 1 protein-interacting protein (PTTG1IP) and DNA (cytosine-5)-methyltransferase 1 (DNMT1) are obviously reduced under ICAM1 knockdown condition, which is consistent with the proteomic result (Fig 6B).

**Table 2:** Down-expressed and p53-related proteins in ICAM1 knockdown CE146T.

Uniprot	Protein Name	Gene Name	ICAM1/shICAM1	M.W. [kDa]	Unique #	PEP
P53007	Tricarboxylate transport protein, mitochondrial	SLC25A1	> 1350	35.05	3	1.97E-09
Q00688	Peptidyl-prolyl cis-trans isomerase FKBP3	FKBP3	> 1105	25.18	2	1.60E-08
Q93009	Ubiquitin carboxyl-terminal hydrolase 7	USP7	> 452	128.30	9	1.49E-09
P53801	Pituitary tumor-transforming gene 1 protein-interacting protein	PTTG1IP	> 362	20.32	2	1.07E-13
O00629	Importin subunit alpha-3	KPNA4	> 350	57.89	4	5.65E-43
P26358	DNA (cytosine-5)-methyltransferase 1	DNMT1	> 253	184.82	4	2.61E-06
Q9Y3F4	Serine-threonine kinase receptor-associated protein	STRAP	> 230	39.78	3	7.53E-25
Q96ST3	Paired amphipathic helix protein Sin3a	SIN3A	> 198	145.17	3	5.55E-01

### ICAM1 and CD44 could utilize a compensation mechanism to maintain esophageal CSC properties

**Fig 7:**
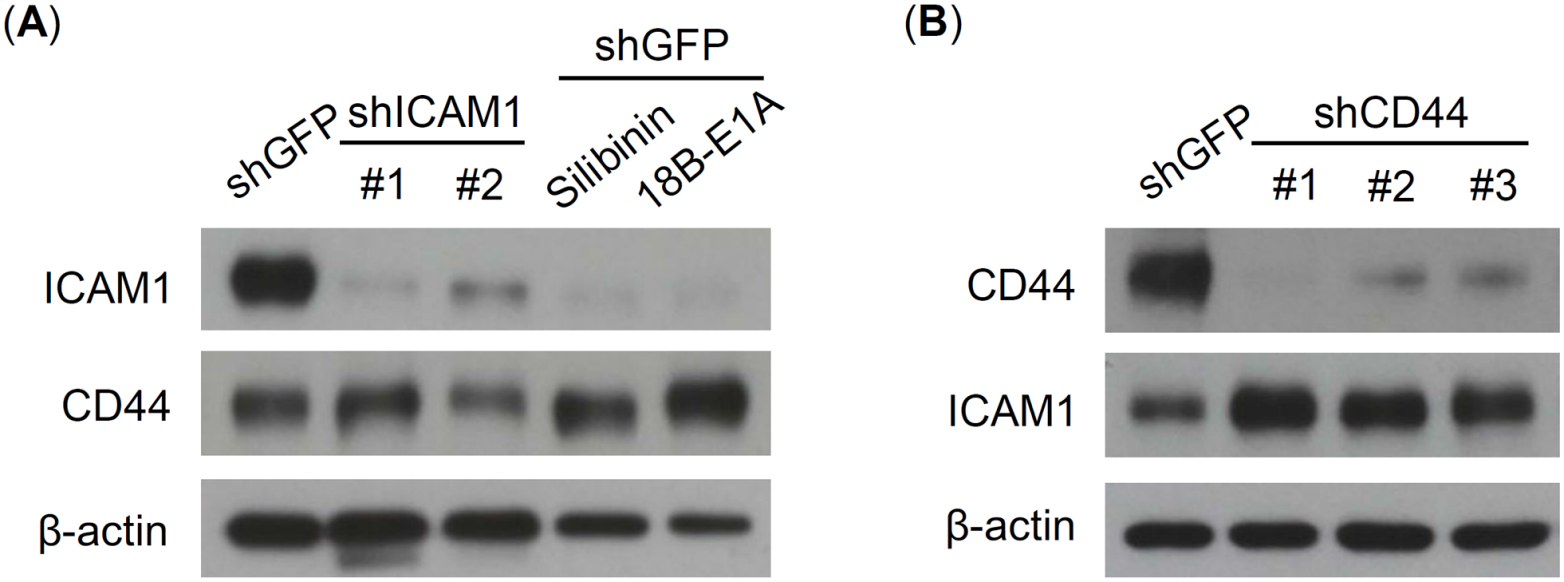
ICAM1 and CD44 compensate their expression in CE146T. ICAM1 and CD44 expression were analyzed using western blot assay. (A) Both shRNA and pharmacological inhibition reduce ICAM1 expression and simultaneously increase CD44 expression. (B) CD44 knockdown leads to increase ICAM1 levels in a compensative manner. CD44 expression was knocked down using three kinds of shRNA, shRNA#1 target sequence is: CGCTATGTCCAGAAAGGAGAA, shRNA#2 target sequence is: ATGGACTCCAGTCATAGTATA, and shRNA#3 target sequence is: GGACCAATTACCATAACTATT. β-actin served as loading control.

In the xenotransplantation experiment, ICAM1 knockdown loses its inhibition power on tumorigenesis at high cell number (10^4^) injection condition (Fig 5B). We wondered whether cross regulation exists in molecules that affect CSC properties, for instance ICAM1 and CD44. To prove this speculation, we reciprocally knocked down ICAM1 and CD44 and determined their ICAM1 and CD44 expression using western blot. The result revealed that ICAM1 knockdown using both shRNA and pharmacological inhibition efficiently reduces ICAM1 expression and simultaneously increases CD44 expression in a reverse correlation manner (Fig 7A). In addition, CD44 knockdown is also able to increase ICAM1 by reverse correlation in a similar manner (Fig 7B). This finding indicated a potential mechanism that cancer cells could use a compensation mechanism to maintain their CSC properties.

## Discussion

Intercellular adhesion molecule 1 (ICAM1, CD54) is a 90 kDa glycosylated transmembrane protein of the immunoglobulin superfamily. ICAM1 plays an important role in immunological synapse formation, T-cell activation, leukocyte trafficking, and numerous cellular immune responses [32]. Previous studies observed that ICAM1 highly expresses in various mesenchymal stem cells including bone marrow, placenta, adipose, periodontal ligament, and Wharton’s jelly [33–36]. Recently, ICAM1 was identified to serve as a marker of hepatocellular carcinoma stem cells [37]. In this study, ICAM1 was identified to express in higher stemness properties of esophageal cancer CE146T cells using a comparative proteomic approach. Our investigation showed the presence of ICAM1 contributes to cancer cell sphere formation, drug resistance, and tumorigenesis in mouse xenotransplantation model, indicating that ICAM1 can be used as a potential CSC marker of ESCC and therefore serve as a therapeutic target for drug design and development.

Systematic meta-analysis pointed out the positive expression of p53 represents a favorable prognostic feature and is consistently associated with overall survival of ESCC [38]. Our study observed that the expression of ICAM1 reversely correlates with p53 levels, suggesting that the high expression of ICAM1 could lead to cancer malignancy. In addition, comparison with proteomic change of CE146T with and without ICAM1 knockdown, we identified a subgroup of proteins related to the expression and functions of p53. Consistent with the proteomic finding, both PTTG1IP and DNMT1 decrease their mRNA levels along with ICAM1 knockdown. PTTG1IP can promote tumor growth and invasion ability, and its high expression is independently associated with poor prognosis and lower disease-specific survival [39]. A recent study demonstrated that PTTG1IP is able to decrease p53 stability by enhancing ubiquitination, which depends on the E3 ligase activity of Mdm2 [40]. This result gives an explanation to our study that ICAM1 knockdown decreases PTTG1IP levels leading to stabilize p53, and thereby increasing p53 levels. DNMT1 is the major enzyme involved in establishing genomic methylation patterns. DNMT1 overexpression was identified in various cancers and it could result in epigenetic alteration of multiple tumor suppressor genes and ultimately lead to tumorigenesis and poor prognosis [41–46]. In addition, DNMT1 overexpression was demonstrated to be associated with loss of repression of p53 in cellular and clinical level [46]. In summary, our observation and the related data suggest a potential mechanism that ICAM1 may regulate CSC properties by an ICAM1-PTTG1IP-p53-DNMT1 pathway. However, this proposed pathway remains to be proved by detailed experiments.

Signaling pathway cross-talk, for instance mTOR/S6K1 and Hedgehog pathways [47] or EGFR and VEGF pathways [48], is well recognized to provide complex interaction and extensive communication in response to cellular stimulation, and lead to synergistic or antagonistic effects and eventually desirable biological outcomes [49]. Whether cancer stem cells use a similar mechanism, molecular cross-talk, to maintain their CSC characteristics remains elusive. In current study, we observed a compensation phenomenon between ICAM1 and CD44 expression levels, which, however, also raises several critical questions. Do ICAM1 and CD44 share common signaling networks to maintain tumor stemness? What is the stoichiometrical relationship between ICAM1 and CD44 in controlling CSC population and properties? Comprehensive investigation will be helpful in revealing the underlying mechanisms involved in the maintenance of ICAM1 and CD44 in CSC of ESCC. Based on such compensation mechanism, our study suggests the combination of multiple targeting strategies should be under consideration for acquiring a more potent therapeutic effect on CSC of ESCC.

## Supporting Information

S1 Table (PDF)
