# Supplementary material for: ICAM1 Is a Potential Cancer Stem Cell Marker of Esophageal Squamous Cell Carcinoma
Source: PLoS One. 2015 Nov 16;10(11):e0142834. doi: 10.1371/journal.pone.0142834 (PMC4646358; doi:10.1371/journal.pone.0142834)
Supplement: S1 Table — (PDF) [file pone.0142834.s001.pdf]

S1 Table. The list of down-expressed proteins in ICAM1 knockdown CE146T.

Uniprot	Protein Name	Gene Name	ICAM1/shICAM1	Unique #	Coverage [%]	MW [kDa]	PEP
Q86Y56	HEAT repeat-containing protein 2	HEATR2	> 1939	2	4.2	93.52	9.75E-29
P53999	Activated RNA polymerase II transcriptional coactivator p15	SUB1	> 1668	2	18.9	14.40	1.87E-34
P28070	Proteasome subunit beta type-4	PSMB4	> 1541	2	7.2	29.20	1.19E-03
P61006	Ras-related protein Rab-8A	RAB8A	> 1450	3	35.3	23.67	3.33E-43
P53007	Tricarboxylate transport protein, mitochondrial	SLC25A1	> 1350	3	10.1	35.05	1.97E-09
Q92922	SWI/SNF complex subunit SMARCC1	SMARCC1	> 1332	4	7.2	122.87	9.14E-64
Q8IYB3	Serine/arginine repetitive matrix protein 1	SRRM1	> 1115	3	5.6	103.80	3.91E-18
Q00688	Peptidyl-prolyl cis-trans isomerase FKBP3	FKBP3	> 1105	2	8.0	25.18	1.60E-08
Q02809	Procollagen-lysine,2-oxoglutarate 5-dioxygenase 1	PLOD1	> 1042	7	9.2	88.27	9.73E-06
P56192	Methionine--tRNA ligase, cytoplasmic	MARS	> 867	5	5.7	101.11	1.47E-29
Q96QD8	Sodium-coupled neutral amino acid transporter 2	SLC38A2	> 779	2	5.3	56.03	2.15E-11
Q9BZZ5	Apoptosis inhibitor 5	API5	> 723	6	9.5	59.00	7.16E-10
P13984	General transcription factor IIF subunit 2	GTF2F2	> 703	2	6.0	28.38	3.70E-02
P49773	Histidine triad nucleotide-binding protein 1	HINT1	> 697	2	12.7	13.80	7.65E-04
Q15785	Mitochondrial import receptor subunit TOM34	TOMM34	> 679	3	7.8	34.56	3.54E-01
Q9GZT3	SRA stem-loop-interacting RNA-binding protein, mitochondrial	SLIRP	> 677	2	17.4	12.35	1.87E-02
P31431	Syndecan-4	SDC4	> 610	2	13.1	21.64	8.40E-22
P29992	Guanine nucleotide-binding protein subunit alpha-11	GNA11	> 555	1	7.2	42.12	1.00E-05
Q9P2B2	Prostaglandin F2 receptor negative regulator [CD315]	PTGFRN	> 549	5	5.2	98.56	1.73E-04
Q9Y490	Talin-1	TLN1	> 547	4	2.0	269.76	1.45E-07
P00846	ATP synthase subunit a	MT-ATP6	> 536	2	6.8	26.05	6.68E-03
Q12904	Aminoacyl tRNA synthase complex-interacting multifunctional protein 1	AIMP1	> 530	2	5.7	37.04	5.28E-18
P14868	Aspartate--tRNA ligase, cytoplasmic	DARS	> 512	5	8.6	57.14	2.30E-10
O94903	Proline synthase co-transcribed bacterial homolog protein	PROSC	> 504	2	8.7	34.04	3.90E-03
Q9Y3I0	tRNA-splicing ligase RtcB homolog	RTCB	> 501	4	7.7	55.21	7.97E-08
P51610	Host cell factor 1	HCFC1	> 486	3	1.7	213.47	3.26E-01
Q9UHG3	Prenylcysteine oxidase 1	PCYOX1	> 483	3	5.5	56.64	3.24E-13
Q00796	Sorbitol dehydrogenase	SORD	> 476	2	7.3	38.32	9.68E-10
Q13501	Sequestosome-1	SQSTM1	> 474	3	9.3	47.69	3.86E-10
P48509	CD151 antigen	CD151	> 470	4	12.3	28.30	2.70E-03
Q03518	Antigen peptide transporter 1	TAP1	> 459	2	3.3	87.18	2.45E-06
Q93009	Ubiquitin carboxyl-terminal hydrolase 7	USP7	> 452	9	7.3	128.30	1.49E-09

Q14498	RNA-binding protein 39	RBM39	> 444	4	8.5	59.38	2.82E-09
O75949	Transmembrane protein FAM155B	FAM155B	> 432	2	4.3	34.47	1.93E-02
P36776	Lon protease homolog, mitochondrial	LONP1	> 405	4	4.0	106.49	1.40E-11
Q16531	DNA damage-binding protein 1	DDB1	> 397	3	2.1	126.97	5.37E-02
P15121	Aldehyde reductase	AKR1B1	> 396	2	5.4	35.85	6.09E-05
P51659	Peroxisomal multifunctional enzyme type 2	HSD17B4	> 393	3	6.0	79.69	3.57E-22
P33947	ER lumen protein retaining receptor 2	KDELR2	> 365	2	9.0	24.42	4.12E-02
P53801	Pituitary tumor-transforming gene 1 protein-interacting protein	PTTG1IP	> 362	2	13.9	20.32	1.07E-13
Q5VTR2	E3 ubiquitin-protein ligase BRE1A	RNF20	> 362	3	3.0	113.66	3.20E-05
O00629	Importin subunit alpha-3	KPNA4	> 350	4	12.1	57.89	5.65E-43
O43175	D-3-phosphoglycerate dehydrogenase	PHGDH	> 349	2	4.9	56.65	2.17E-13
P39748	Flap endonuclease 1	FEN1	> 342	4	8.7	42.59	1.28E-03
Q14CM0	FERM and PDZ domain-containing protein 4	FRMPD4	> 338	2	1.2	144.38	1.44E-01
Q13492	Phosphatidylinositol-binding clathrin assembly protein	PICALM	> 333	2	3.7	70.75	2.23E-03
Q02338	D-beta-hydroxybutyrate dehydrogenase, mitochondrial	BDH1	> 325	2	4.4	38.16	2.83E-03
Q99460	26S proteasome non-ATPase regulatory subunit 1	PSMD1	> 323	3	4.7	105.84	3.29E-03
P11413	Glucose-6-phosphate 1-dehydrogenase	G6PD	> 301	4	6.4	63.83	6.63E-02
O15427	Monocarboxylate transporter 4	SLC16A3	> 289	2	4.5	49.47	3.59E-05
P82933	28S ribosomal protein S9, mitochondrial	MRPS9	> 275	3	6.6	45.83	1.76E-11
Q14739	Lamin-B receptor	LBR	> 267	4	4.6	70.70	2.12E-02
O14776	Transcription elongation regulator 1	TCERG1	> 257	4	3.6	123.90	3.56E-05
P06756	Integrin alpha-V [CD51]	ITGAV	> 254	4	3.0	116.04	1.67E-02
P13674	Prolyl 4-hydroxylase subunit alpha-1	P4HA1	> 253	3	4.7	61.05	8.34E-05
P26358	DNA (cytosine-5)-methyltransferase 1	DNMT1	> 253	4	3.4	184.82	2.61E-06
Q5JTH9	RRP12-like protein	RRP12	> 251	2	1.8	143.70	2.97E-09
Q9BUQ8	Probable ATP-dependent RNA helicase DDX23	DDX23	> 248	2	2.4	95.58	5.48E-04
Q8WXH0	Nesprin-2	SYNE2	> 246	2	0.2	798.85	7.41E-02
P28340	DNA polymerase delta catalytic subunit	POLD1	> 235	3	3.8	126.39	8.35E-05
O00154	Cytosolic acyl coenzyme A thioester hydrolase	ACOT7	> 233	2	7.0	40.46	2.67E-38
Q9Y3F4	Serine-threonine kinase receptor-associated protein	STRAP	> 230	3	9.9	39.78	7.53E-25
Q9NVA2	Septin-11	SEPT11	> 222	2	7.7	50.82	2.78E-05
Q9UK59	Lariat debranching enzyme	DBR1	> 216	2	3.5	61.55	2.20E-04
O43290	U4/U6.U5 tri-snRNP-associated protein 1	SART1	> 214	3	4.0	90.25	1.77E-12
Q14151	Scaffold attachment factor B2	SAFB2	> 213	5	6.7	102.85	6.63E-24
Q15293	Reticulocalbin-1	RCN1	> 213	2	5.7	38.89	6.07E-18

Q9NSD9	Phenylalanine--tRNA ligase beta subunit	FARSB	> 209	2	3.7	66.12	6.27E-12
Q96QK1	Vacuolar protein sorting-associated protein 35	VPS35	> 208	3	4.3	91.71	1.77E-24
P11387	DNA topoisomerase 1	TOP1	> 207	2	2.6	90.73	3.69E-08
Q96ST3	Paired amphipathic helix protein Sin3a	SIN3A	> 198	3	2.1	145.17	5.55E-01
P50995	Annexin A11	ANXA11	> 192	2	3.0	65.60	2.20E-10
Q99536	Synaptic vesicle membrane protein VAT-1 homolog	VAT1	> 191	2	2.8	41.92	4.49E-03
P08962	CD63 antigen	CD63	> 185	2	6.7	25.64	1.43E-03
Q7Z2K6	Endoplasmic reticulum metallopeptidase 1	ERMP1	> 183	2	1.8	100.23	1.83E-02
O43747	AP-1 complex subunit gamma-1	AP1G1	> 180	3	5.3	94.14	1.13E-34
Q9UGP8	Translocation protein SEC63 homolog	SEC63	> 171	2	2.6	88.00	1.45E-06
Q9Y6M1	Insulin-like growth factor 2 mRNA-binding protein 2	IGF2BP2	> 158	3	4.6	66.79	1.80E-04
Q92900	Regulator of nonsense transcripts 1	UPF1	> 157	3	3.1	124.34	1.92E-02
P46060	Ran GTPase-activating protein 1	RANGAP1	> 153	2	3.7	63.54	1.94E-12
O75150	E3 ubiquitin-protein ligase BRE1B	RNF40	> 152	3	2.2	113.65	1.95E-02
Q8N1F7	Nuclear pore complex protein Nup93	NUP93	> 149	3	3.7	93.49	8.25E-29
Q9BUJ2	Heterogeneous nuclear ribonucleoprotein U-like protein 1	HNRNPUL1	> 148	2	2.5	95.74	3.19E-02
O75874	Isocitrate dehydrogenase [NADP] cytoplasmic	IDH1	> 147	2	5.8	46.66	4.04E-05
Q7Z6Z7	E3 ubiquitin-protein ligase HUWE1	HUWE1	> 136	4	0.9	482.14	1.89E-07
P30419	Glycylpeptide N-tetradecanoyltransferase 1	NMT1	> 121	2	4.4	56.81	1.33E-03
Q14247	Src substrate cortactin	CTTN	> 110	2	2.7	70.96	1.57E-02
Q08345	Epithelial discoidin domain-containing receptor 1 [CD167a]	DDR1	> 109	2	2.1	101.80	3.28E-02
Q9HAV4	Exportin-5	XPO5	> 63	2	1.4	136.31	8.91E-02
P54577	Tyrosine--tRNA ligase, cytoplasmic	YARS	58.2	5	9.3	59.14	3.82E-13
P01024	Complement C3	C3	46.6	15	10.2	187.15	1.88E-126
Q9Y295	Developmentally-regulated GTP-binding protein 1	DRG1	26.3	3	11.2	40.54	3.39E-86
O95197	Reticulon-3	RTN3	25.9	2	5.6	112.61	5.79E-48
Q9Y320	Thioredoxin-related transmembrane protein 2	TMX2	23.4	3	8.1	42.47	1.87E-02
P11498	Pyruvate carboxylase, mitochondrial	PC	23.3	4	4.5	129.63	7.28E-03
O43719	HIV Tat-specific factor 1	HTATSF1	15.9	6	6.8	85.85	9.10E-69
P16422	Epithelial cell adhesion molecule [CD326]	EPCAM	15.2	5	12.6	37.89	7.84E-34
P47897	Glutamine--tRNA ligase	QARS	14.7	3	4.6	87.80	3.10E-06
P49821	NADH dehydrogenase [ubiquinone] flavoprotein 1, mitochondrial	NDUFV1	14.2	3	6.7	50.82	4.10E-03
P35249	Replication factor C subunit 4	RFC4	13.4	4	12.4	39.68	3.48E-08
P69849	Nodal modulator 3	NOMO3	13.1	3	2.8	139.44	2.17E-08
P54136	Arginine--tRNA ligase, cytoplasmic	RARS	12.3	3	3.8	75.38	1.13E-07

Q92616	Translational activator GCN1	GCN1L1	12.0	5	1.8	292.75	1.68E-07
P05362	Intercellular adhesion molecule 1 [CD54]	ICAM1	11.8	4	10.0	57.83	1.38E-06
